# β-Adrenoceptor blockade prevents carotid body hyperactivity and elevated vascular sympathetic nerve density induced by chronic intermittent hypoxia

**DOI:** 10.1007/s00424-020-02492-0

**Published:** 2020-11-19

**Authors:** Abdulaziz A. Alzahrani, Lily L. Cao, Hayyaf S. Aldossary, Demitris Nathanael, Jiarong Fu, Clare J. Ray, Keith L. Brain, Prem Kumar, Andrew M. Coney, Andrew P. Holmes

**Affiliations:** 1 Institute of Clinical Sciences, University of Birmingham, Edgbaston, Birmingham, B15 2TT UK; 2 Respiratory Care Department, Faculty of Applied Medical Sciences, Umm Al-Qura University, Makkah, Saudi Arabia; 3 Institute of Cardiovascular Sciences, University of Birmingham, Edgbaston, Birmingham, B15 2TT UK; 4 College of Medicine, Basic Medical Sciences, King Saud bin Abdulaziz University for Health Sciences, Riyadh, Saudi Arabia

**Keywords:** Carotid body, Hypoxia, Adrenaline, β-Adrenoceptors, Chronic intermittent hypoxia, Hypertension, Beta-blockers, Vascular sympathetic nerves

## Abstract

Carotid body (CB) hyperactivity promotes hypertension in response to chronic intermittent hypoxia (CIH). The plasma concentration of adrenaline is reported to be elevated in CIH and our previous work suggests that adrenaline directly activates the CB. However, a role for chronic adrenergic stimulation in mediating CB hyperactivity is currently unknown. This study evaluated whether beta-blocker treatment with propranolol (Prop) prevented the development of CB hyperactivity, vascular sympathetic nerve growth and hypertension caused by CIH. Adult male Wistar rats were assigned into 1 of 4 groups: Control (N), N + Prop, CIH and CIH + Prop. The CIH paradigm consisted of 8 cycles h^−1^, 8 h day^−1^, for 3 weeks. Propranolol was administered via drinking water to achieve a dose of 40 mg kg^−1^ day^−1^. Immunohistochemistry revealed the presence of both β_1_ and β_2_-adrenoceptor subtypes on the CB type I cell. CIH caused a 2–3-fold elevation in basal CB single-fibre chemoafferent activity and this was prevented by chronic propranolol treatment. Chemoafferent responses to hypoxia and mitochondrial inhibitors were attenuated by propranolol, an effect that was greater in CIH animals. Propranolol decreased respiratory frequency in normoxia and hypoxia in N and CIH. Propranolol also abolished the CIH mediated increase in vascular sympathetic nerve density. Arterial blood pressure was reduced in propranolol groups during hypoxia. Propranolol exaggerated the fall in blood pressure in most (6/7) CIH animals during hypoxia, suggestive of reduced sympathetic tone. These findings therefore identify new roles for β-adrenergic stimulation in evoking CB hyperactivity, sympathetic vascular hyperinnervation and altered blood pressure control in response to CIH.

## Introduction

The carotid body (CB) is the major sensory organ in the human body that responds to acute hypoxic stress [30, 50]. When stimulated the CB activates vital protective reflexes including hyperventilation, systemic vasoconstriction and elevated heart rate [29, 30]. CB activation in hypoxia is critical to maintain adequate O_2_ delivery to the brain and vital organs. However, when exposed to chronic intermittent hypoxia (CIH) the CB becomes pathologically hyperactive leading to chronic reflex activation and cardiovascular disease. It is now clear that CB hyperactivity is important for hypertension development in patients with obstructive sleep apnoea (OSA) who are exposed to nightly episodes of CIH [14, 23, 44, 57, 58]. CB hyperactivity also promotes cardiac arrhythmias, hypertension and ventilatory dysfunction in animals exposed to CIH [8, 52, 55], validating this as a robust model to explore causes of CB hyperactivity and cardiovascular disease. However, the mechanisms underpinning CB hyperactivity caused by CIH are unresolved and there are currently no specific drug therapies used clinically that directly target the CB. In addition to modifications in CB function, emerging evidence suggests that CIH increases sympathetic vascular nerve growth [34]. As yet, relatively little is known about the mechanisms accounting for this vascular hyperinnervation and if this can be prevented by pharmacological intervention.

We have recently identified that adrenaline (Adr) is a physiological activator of the CB [72]. CB stimulation by Adr during hypoglycaemia elevates ventilation that is needed to match a rise in metabolic rate and maintain blood pH [72]. The physiological activation of the CB by Adr can be blocked by propranolol [72], implicating an important role for β-adrenoceptors. However, it is not yet known which β-adrenoceptor subtypes are expressed in the CB. Interestingly, exposure to CIH leads to a chronic rise in plasma Adr concentration in both adults and children [13, 26, 37]. These findings have been verified in animals exposed to CIH [56, 59]. Our previous work does suggest that in addition to causing hypertension, chronic exogenous application of catecholamines also enhance the hypoxic ventilatory response, consistent with CB hyperactivity [15]. However, it is not known if chronic endogenous Adr contributes to CB hyperactivity caused by CIH [22]. Furthermore, a role for chronic adrenergic stimulation in mediating vascular nerve growth during CIH is yet to be determined.

This study therefore investigated whether beta-blocker treatment with propranolol was able to prevent the development of CB hyperactivity, vascular sympathetic hyperinnervation and neurogenic hypertension induced by CIH.

## Methods

### Ethical approval

All procedures were performed in accordance with UK Animals (Scientific Procedures) Act 1986, and approved by the UK Home Office (PPL number PF4C074AD) and by the Animal Welfare and Ethical Review Body (AWERB) at the University of Birmingham. Adult male Wistar rats (*n* = 69, 9–10 weeks) were purchased from Charles River, UK. Animals were housed in individually ventilated cages (*n* = 2–4 per cage) under standard conditions: 12:12 h light:dark cycle (lights on at 0700), 22 °C and 55% humidity. Food and water were available ad libitum. Terminal experiments (described below) were performed on animals aged 14–15 weeks, weight range 350–500 g. To reduce animal numbers, where possible the same animal was used for recovery and terminal in vivo experiments (e.g. plethysmography and cardiovascular measurements) or for in vivo and ex vivo experiments (e.g. plethysmography and isolated CB). In these cases, experiments were performed on separate days to avoid any potential crossover effects.

### Experimental design, administration of propranolol and induction of CIH

Animals were assigned randomly by cage into 1 of 4 experimental groups: normal ambient air (N; *n* = 21), normal ambient air treated with propranolol (N + Prop; *n* = 17), exposure to CIH (CIH; *n* = 16) and exposure to CIH with propranolol treatment (CIH + Prop; *n* = 15). For the N + Prop and CIH + Prop, propranolol was administered via drinking water to achieve doses of 40 mg kg^−1^ day^−1^ for 10 days prior to and during CIH. The criteria for preparing the propranolol in drinking water was based on the average daily water consumption of the animals, the targeted drug dose [35] and total animal weight in the cage. This was monitored twice a week and adjusted if needed. For CIH and CIH + Prop animals, hypoxia cycles were applied for 8 h per day (8:00 am to 4:00 pm), 7 days a week for 3 weeks using an OxyCycler A410V dynamic O_2_ controller and dedicated small animal chambers (BioSpherix, Parish, NY, USA). A total of 64 cycles of hypoxia were delivered to the animals per day (8 cycles per hour). The animals were exposed to a slow reduction from 21% O_2_ to a nadir of 5% O_2_ over 150 s. The O_2_ was held at 5% O_2_ for 15 s before returning to 21% O_2_. Throughout the CIH procedure, animals were housed in groups of two, were able to move freely and food and water was available ad libitum. After each day of CIH induction, animals were returned to their home cage.

### Extracellular recordings of CB sensory activity

Intact carotid bifurcations containing the carotid sinus nerve (CSN) and CB were removed from adult male Wistar rats (350–550 g, *n* = 29) under deep inhalation anaesthesia (2.5–5% isoflurane in O_2_, 1.5 L min^−1^). After tissue removal, animals were immediately killed by cervical dislocation. To aid extracellular neuronal recordings, the whole tissue was partially digested by incubation in enzyme Krebs solution (collagenase type II, 0.075 mg/mL (C6885) and dispase type I; 0.0025 mg/mL (D4818, Sigma Aldrich, Gillingham, UK), at 37 °C, for 30 min. The CB preparation was subsequently placed in a recording chamber and was continuously superfused with a standard bicarbonate buffered Krebs solution containing, in mM: 115 NaCl, 4.5 KCl, 1.25 NaH_2_PO_4_, 1.3 MgSO_4_, 24 NaHCO_3_, 2.4 CaCl_2_ and 11 D-glucose, 37 °C, pH 7.4. The superfusate PO_2_ was measured using an O_2_ electrode (ISO2) and O_2_ meter (OXELP; World Precision Instruments, Hitchin, UK) and sampled at 100 Hz. Extracellular recordings of CB sensory activity were recorded from the cut end of the CSN using borosilicate glass pipettes [17, 21]. Raw chemoafferent voltage was amplified × 4000, band-pass filtered between 50 Hz and 50 kHz and digitized at 15 kHz using a CED micro1401 (Cambridge Electronic Design, Cambridge, UK). Acquisition and analysis were performed using Spike2 (version 7.12) software (Cambridge Electronic Design, Cambridge, UK). Single fibres were used for frequency analysis. Action potentials (APs) originating from a single sensory fibre was determined based on a unique AP waveform shape and amplitude [17, 21].

Basal single fibre AP frequency was measured in the standard Krebs solution equilibrated with a PO_2_ of 300 mmHg and PCO_2_ of 40 mmHg. This PO_2_ has been shown to generate a basal frequency consistent with that measured in vivo in the rat in arterial normoxia [17, 74]. To generate peak responses to hypoxia, the superfusate PO_2_ was gradually reduced using high precision flow meters (Cole-Parmer Instrument Company, St. Neots, UK) at constant PCO_2_, to achieve a bath PO_2_ of *ca* 40 mmHg. This stimulus was applied for 5 min to monitor the ability of the CB to sustain the response. The sustained response was taken as the mean frequency recorded in the final 60 s of the hypoxic stimulus. To evaluate chemoafferent responses to mitochondrial inhibition, sodium nitrite (Na_2_NO_2_, 10 mM, osmolality balanced with reduced NaCl) was used to induce moderate elevations in chemoafferent discharge at a bath PO_2_ = 300 mmHg and PCO_2_ = 40 mmHg [19, 21]. Nitrite was used at a concentration previously shown to elevate NADH autofluorescence in CB type I cells, consistent with mitochondrial inhibition [19]. It was used in this study as the response is rapid and readily reversible without causing persistent damage to the CB [19]. Steady state responses were taken from the final 60 s of a 5-min application. Responses to hypercapnia were induced by increasing the PCO_2_ to 80 mmHg [18, 46]. The steady state responses were taken from the final 60 s of a 5-min hypercapnic exposure. As the response to CO_2_ is linear over this range, the CO_2_ sensitivity can be calculated as the Δ Hz per mmHg rise in PCO_2_.

### Vascular sympathetic innervation density

2nd or 3rd order mesenteric arteries (MAs) were harvested from 14 animals (N, *n* = 4; CIH, *n* = 5; CIH + Prop, n = 5) after confirmation of death following cervical dislocation under deep inhalation anaesthesia (2.5–5% isoflurane in O_2_, 1.5 L min^−1^). MAs were transferred to an imaging chamber and loaded with a fluorescent dye (Neurotransmitter Transporter Uptake Assay, NTUA; Molecular Devices, CA, USA) via a superfusion delivery system (flow rate: 2 mL min^−1^) to reveal noradrenergic nerves. NTUA is a substrate for the prejunctionally located noradrenaline transporter (NAT); NTUA becomes unmasked and fluoresces upon entry into rodent periarterial sympathetic nerve terminals [6]. Z-stacks (512 × 512 pixels, ~ 60 slices at 1 μm interval) were taken with an upright confocal scanning microscope (Olympus Fluoview FV1000, Tokyo, Japan) using a 40× 1.3 NA oil immersion objective with wavelength filter set at: excitation 405 nm and emission 460–560 nm. Per animal, one vessel was loaded with NTUA and three different areas of the vessel were imaged. Z-stacks were compiled on Fiji (version 1.52a; https://fiji.sc) and subjected to nerve density calculation as described previously [49, 66]. In brief, fluorescence intensities three standard deviations above the mean background signal were identified as noradrenergic nerve labelling; this was expressed as a percentage of nerve fibres to total vascular area. Nerve intercepts of each image were counted on three different days. Surface density was calculated by superimposing a grid of horizontal- and vertical lines at 25 μm intervals and counting the total number of times individual sympathetic nerve fibres intersected the grid lines; nerve density was expressed as nerve intercepts per μm tissue. We also utilised NTUA to assess single-terminal NAT reuptake function, an optical technique validated in rodent periarterial nerves ex vivo [6]; tissues were initially superfused with 1:100 NTUA dilution to identify nerve terminals, followed by 1:20 NTUA with frequent image acquisition. Changes in fluorescence during the latter period was measured in individual nerve terminals and the rate was presented as the increase over time, normalised to the starting 1:100 NTUA fluorescence to allow for comparison between experiments.

### Immunohistochemistry

An additional 3 control (N; *n* = 3) animals were used to evaluate the specific expression of β_1_ and/or β_2_-adrenoceptors in the CB. Carotid bifurcations, including the CB, were removed under inhalation anaesthesia with 2.5–5% isoflurane in O_2_ (flow rate 1.5 L min^−1^). CBs were immediately fixed in 4% paraformaldehyde in 10 mM PBS, pH 7.4, for 2 h and then placed in a 30% sucrose solution for 24 h. The tissue was then embedded in OCT compound (TAAB Laboratories, Aldermaston, UK), frozen and stored at − 80 °C. CB tissue was sectioned at a thickness of 10 μm and adhered onto charged microscope slides (HistoBond®+S, VWR International, Lutterworth, UK). Sections were washed in PBS, permeabilised in PBS containing 0.1% Triton X-100 and blocked for 30 min in PBS containing 1% BSA and 0.05% Tween20. Mouse monoclonal anti-tyrosine hydroxylase (TH; 1:20) antibodies (Abcam Ltd., Cambridge, UK) were used to positively identify type I cells in the CB [17]. Goat monoclonal anti-β_1_ (1:50, 1:250) or rabbit monoclonal anti-β_2_ (1:50, 1:250) adrenoceptor antibodies (Abcam Ltd., Cambridge, UK) were used to label adrenoceptors. Primary antibodies were diluted in PBS containing 0.1% BSA, 0.1% Tween20, applied to the sections and incubated in a humidified chamber at 4 °C for 24 h. Sections were washed 3x in PBS containing 0.1% Tween20 to remove excess primary antibodies. The sections were then incubated in PBS containing 0.1% BSA, 0.1% Tween20 and anti-mouse Alexa Fluor 594 (1:250) with either anti-goat Alexa Fluor 488 (1:250) or anti-rabbit Alexa Fluor 488 (1:250; Molecular Probes, Paisley UK) in a dark humidified chamber for 2 h. Sections were mounted with antifade mounting medium containing DAPI (Vector Laboratories Ltd., UK) and cover-slipped. Sections were visualised using a confocal microscope (Olympus Fluoview FV1000, Tokyo, Japan) with 10× 0.4 NA objective to initially locate the CB and then 40× oil immersion objective to image type I cell clusters. At 40× magnification, images were acquired at 512 × 512 pixel resolution, one way mode at 2 μs pixel^−1^, pinhole size at 85 μm, and line averaging of 8. Excitation wavelengths were 559 nm (laser power: 4%) and 488 nm (laser power: 3%) and emission filters were 570–670 nm and 500–545 nm, respectively. Image merging was performed using Fiji.

### Whole-body plethysmography

In vivo baseline ventilation and the response to hypoxia was assessed in freely moving awake animals (*n* = 34) using a whole-body plethysmography (WBP) system (Emka Technologies, Paris, Fr). This is a non-invasive method to record standard respiratory function including tidal volume (V_t_), respiratory frequency (R_f_) and minute ventilation (V_E_). Respiratory signals were acquired and analysed using iox2 software (Emka Technologies, Paris, Fr). Delivery of the desired hypoxic/hypercapnic gas mixtures was performed using high precision mass flow controllers driven by iox2 (Emka Technologies, Paris, Fr). All experiments were carried out between (09:00–13:00). The protocol consists of 30 min of ventilation in normoxia (21% F_i_O_2_) to acclimatise the animal to the new environment. For hypoxia, the F_i_O_2_ was decreased gradually to 10% and ventilation was recorded over 5 min with a mean of the final 2 min taken for analysis. Artefacts induced by animal movement as well as sigh events were excluded from the analysis. The animals were weighed before the experiments to normalise V_t_ and V_E_ to body mass.

### In vivo cardiovascular responses to hypoxia

Adult male Wistar rats (*n* = 32) were initially anaesthetised with 3–5% isoflurane in O_2_ at 3–4 L min^−1^ (Merial Animal Health Ltd., UK). Following cannulation of the right femoral vein, isoflurane was removed and anaesthesia was maintained with i.v. alfaxalone (Alfaxan®; Vétoquinol UK Ltd), at 17–20 mg kg^−1^ h^−1^ with 0.1 mL boluses as necessary [21, 72]. Core body temperature was maintained at 37 °C with a surgical table heating system. The right femoral artery was cannulated and connected to a physiological pressure transducer (ADInstruments, Oxford, UK) to monitor arterial blood pressure (ABP) and to derive the heart rate (HR). The trachea was cannulated and a spirometer was attached to measure air flux. Animals were allowed to stabilize for 20 min following surgery. Hypoxia was administered over 5 min (F_i_O_2_ 10% balance with N_2_). The HR and mean arterial blood pressure (MABP) responses to hypoxia were taken as the mean of the final 2 min of the hypoxic exposure. Baseline HR and MABP were taken as the mean of the last 5 min before inducing hypoxia. Data were recorded using PowerLab and Labchart software (ADInstruments, Oxford, UK). At the end of the experiment, animals were killed by overdose of sodium pentobarbital (Euthatal®; 200 mg mL^−1^, Merial Animal Health Ltd), confirmed by cervical dislocation.

### Analysis of data

Individual data points represent averaged data from a single animal. All individual data points and the median are presented in box-whisker plots, where the box limits indicate the inter-quartile range and the whiskers extend to the outliers. Statistical analysis was performed using a two-way Analysis of Variance (ANOVA), unless otherwise stated, to evaluate overall effects of CIH and propranolol, and any interaction between these variables (GraphPad Prism 8, GraphPad Prism Software, San Diego, CA, USA). Tukey or Bonferroni post hoc analysis was performed where appropriate to identify specific effects of propranolol in N and CIH animals. Significance was taken as *p* < 0.05. Data is presented in the text as mean ± SD.

## Results

### β_1_ and β_2_-adrenoceptors are expressed in the adult CB type I cell

**Fig. 1:**
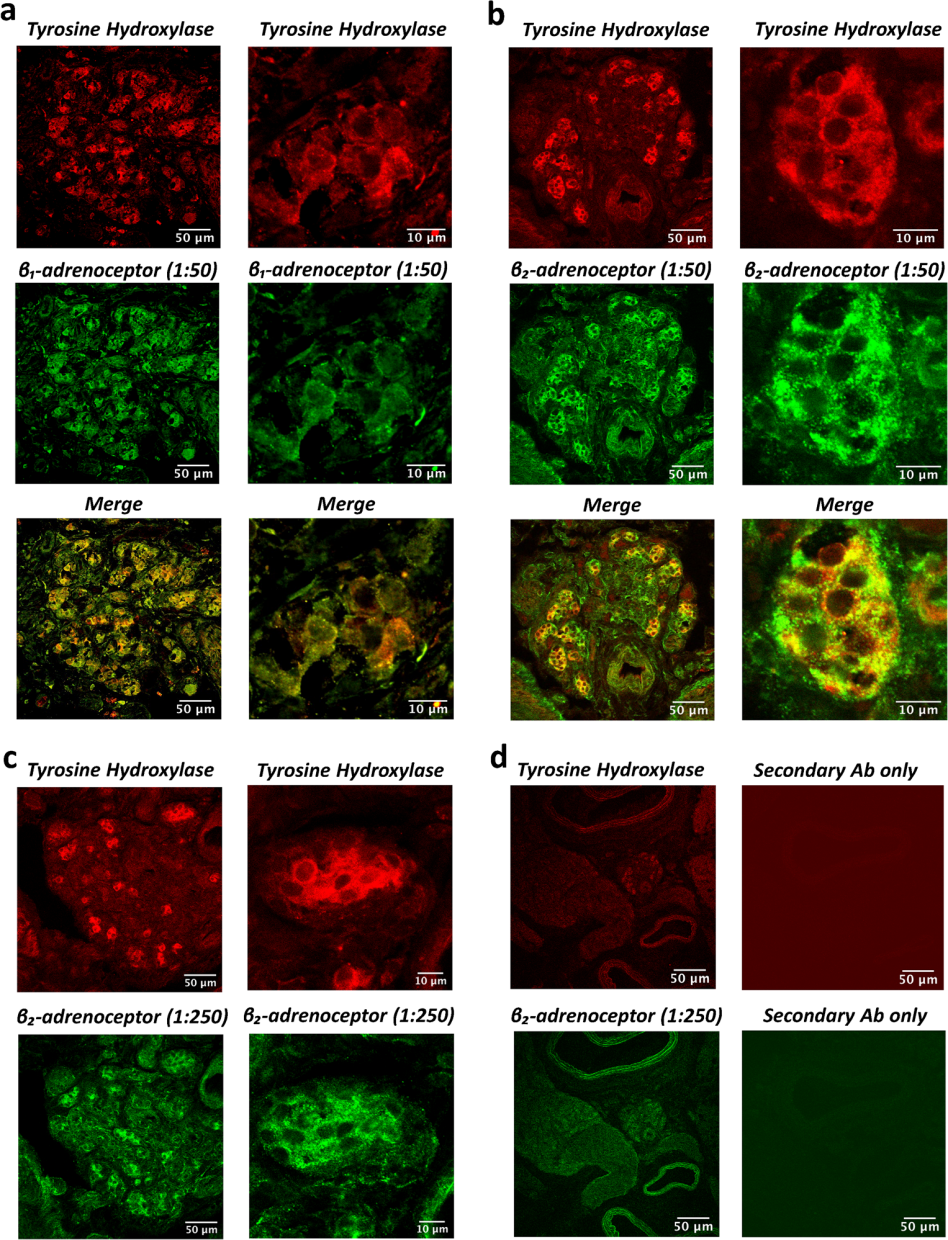
β_1_ and β_2_-adrenoceptors are expressed in the adult carotid body (CB) type I cell. Immunohistochemistry staining of CB tissue sections (10 μm) showing co-localisation of both **a** β_1_ and **b** β_2_-adrenoceptors in the CB (scale bar = 50 μm; low magnification, scale bar = 10 μm; high magnification). Top images (red) demonstrate staining for tyrosine hydroxylase (TH, 1:20 primary Ab dilution), a marker of CB type I cells. Middle images (green) show staining of both β_1_-adrenoceptors (left, 1:50) and β_2_-adrenoceptors (right, 1:50). Bottom images are merged to reveal co-localisation of both β_1_- and β_2_-adrenoceptors in TH positive CB type I cells. Staining was consistent in multiple sections from *n* = 3 control (N) animals. **c** Top images (red) demonstrate staining for TH (1:20 primary Ab dilution) and below images (green) show staining of β_2_-adrenoceptors at a lower Ab dilution (1:250). **d** Positive identification of TH (1:20) and β_2_-adrenoceptors (1:50) in the CB (left column). When incubated with secondary antibodies (Ab) only (Alexa Fluor 594 (1:250) and Alexa Fluor 488 (1:250)) minimal background signal was detected (right column)

Experiments were carried out to identify potential protein expression of β_1_ and β_2_-adrenoceptor subtypes in the CB. Figure 1 shows positive identification of both β_1_ (Fig. 1a) and β_2_-adrenoceptors (Fig. 1b; green) in the CB. Both receptors demonstrate strong co-localisation with the positive marker for type I cells (tyrosine hydroxylase; red). This is apparent when viewed across a section of the whole CB or in a single cluster containing type I cells (Fig. 1a and b). β_1_ and β_2_-adrenoceptors are not homogenously distributed and there are some indications of receptor clustering or hotspots as evidenced by areas of increased fluorescence intensity. Thus, both β_1_ and β_2_-adrenoceptors subtypes are expressed on the type I cell of the CB. However, there are also regions in the CB that show expression of β_1_ and β_2_-adrenoceptors not co-localised with type I cells, possibly suggestive of the presence of these receptors in other structures. Positive staining of both β-adrenoceptors was reproducible in multiple CB sections from three control (N) animals. Furthermore, a positive signal for β_2_-adrenoceptors could still be detected at a lower primary Ab dilution of 1:250, again exhibiting co-localisation with tyrosine hydroxylase positive CB type I cells (Fig. 1c). Sections stained with secondary Ab only produced minimal fluorescent signals, consistent with a low level of non-selective binding (Fig. 1d).

### Chronic propranolol treatment reduces baseline CB hyperactivity induced by CIH

**Fig. 2:**
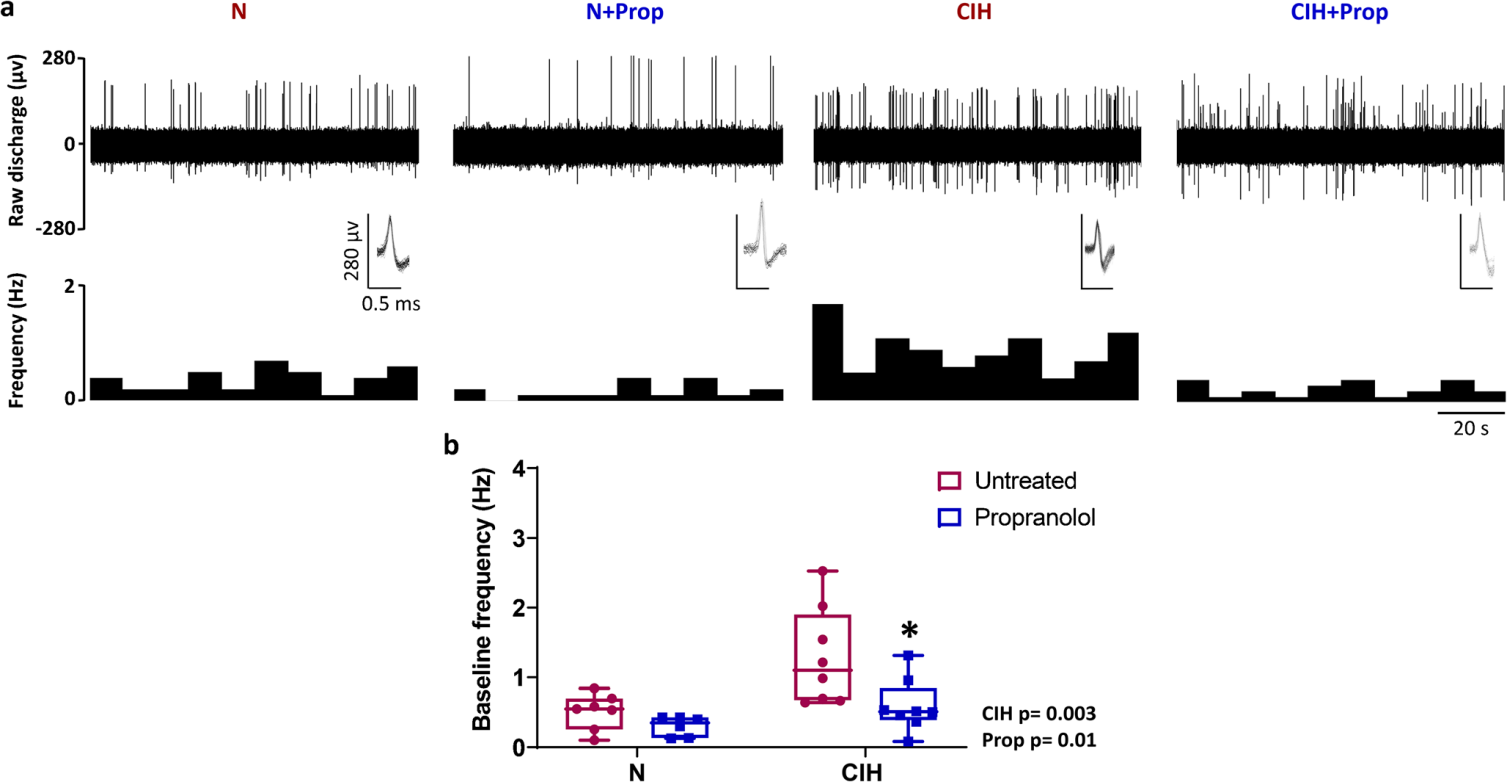
Chronic propranolol treatment reduces baseline carotid body (CB) hyperactivity caused by chronic intermittent hypoxia (CIH). **a** Characteristic examples of raw ex vivo baseline CB sensory nerve activity recorded in normoxia (300 mmHg PO_2_) for N, N + Prop, CIH and CIH + Prop animals. Raw nerve activity is shown (upper) along with frequency histograms (lower). Inset: multiple overdrawn action potentials are shown to exhibit single fibre discrimination. **b** Mean baseline single fibre CB frequency for N (*n* = 7), N + Prop (*n* = 6), CIH (*n* = 8) and CIH + Prop (n = 8) animals. Individual points represent averaged data for a single animal. Data are presented as box and whisker plots with median; the 25th and 75th percentiles form the box and whiskers extend to outliers. Overall effects of CIH and propranolol are shown as text and * denotes *p* ˂ 0.05 CIH vs CIH + Prop; two-way ANOVA with Tukey’s multiple-comparisons test

Measurements of chemoafferent activity were made to assess the impact of CIH and chronic propranolol treatment on CB function. Example recordings are shown in Fig. 2a and demonstrate that baseline chemoafferent hyperactivity caused by 3 weeks of CIH is abolished by propranolol treatment. Mean data suggests that CIH leads to an approximately 2 to 3-fold elevation in baseline activity (Fig. 2b). Propranolol treatment reduced baseline CB activity and post hoc analysis indicates that this is more apparent in CBs isolated from animals exposed to CIH (Fig. 2b). Propranolol treatment during CIH exposure attenuated the development of baseline hyperactivity, with 5/7 animals displaying chemoafferent activity very similar to normoxic controls (Fig. 2b).

### Chronic propranolol treatment attenuates CB responses to hypoxia, mitochondrial inhibition and hypercapnia

**Fig. 3:**
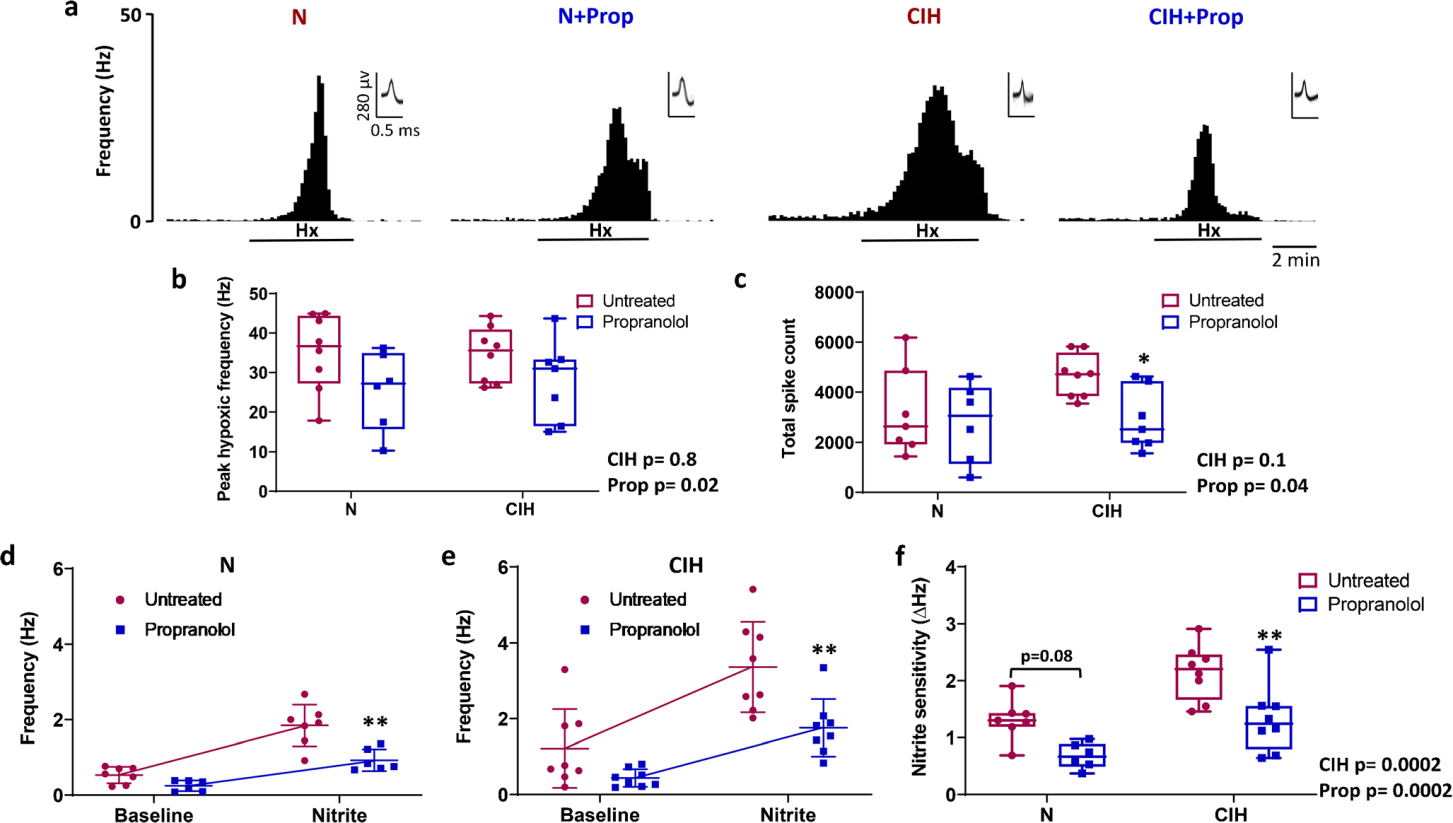
Chronic propranolol treatment attenuates carotid body (CB) chemoafferent responses to hypoxia and mitochondrial inhibition. **a** Examples of ex vivo CB chemoafferent frequency responses to 5 min of hypoxia (PO_2_ of *ca* 40 mmHg) for N, N + Prop, CIH and CIH + Prop animals. Frequency histograms (lower) are shown along with multiple overdrawn action potentials (inset) to demonstrate single fibre discrimination. **b** Mean peak frequency responses to hypoxia for N (*n* = 7), N + Prop (*n* = 6), CIH (*n* = 7) and CIH + Prop (*n* = 8) animals. **c** Total number of action potentials (spikes) recorded throughout the entire 5 min of hypoxic exposure for each of the 4 groups. **d**, **e** Mean discharge frequency measured at baseline and in response to 10 mM nitrite (a mitochondrial inhibitor) in N (*n* = 7), N + Prop (*n* = 6), CIH (*n* = 8) and CIH + Prop (*n* = 8) animals. **f** Mean nitrite sensitivity (nitrite-baseline) for all 4 groups. Individual points represent averaged data for a single animal. For b, c & f, data are presented as box and whisker plots with median; the 25th and 75th percentiles form the box and whiskers extend to minimum and maximum values. Overall effects of CIH and Propranolol are shown as text and *, ** denotes *p* < 0.05, *p* ˂ 0.01 CIH vs CIH + Prop respectively; two-way ANOVA with Tukey’s multiple-comparisons test. For d and e, data presented as mean ± SD. ** denotes *p* < 0.01 Propranolol vs untreated for N and CIH animals; two-way repeated measures ANOVA with Bonferroni multiple-comparisons test

We also measured peak and sustained chemoafferent responses to hypoxia, nitrite (NO_2_^−^; a mitochondrial inhibitor) and hypercapnia. Propranolol attenuated the peak chemoafferent activity in hypoxia by approximately 25–30% in both N and CIH animals (Fig. 3a and b). Propranolol also significantly reduced the total chemoafferent spike count in CIH animals when measured throughout the entire 5 min of sustained hypoxic exposure (Fig. 3c). Propranolol treatment decreased chemoafferent frequency in the presence of nitrite in both N and CIH animals (Fig. 3d and e). However, nitrite sensitivity (nitrite-baseline) was only significantly reduced in CIH + Prop, suggestive of a greater effect of propranolol in CIH animals (Fig. 3f).

**Fig. 4:**
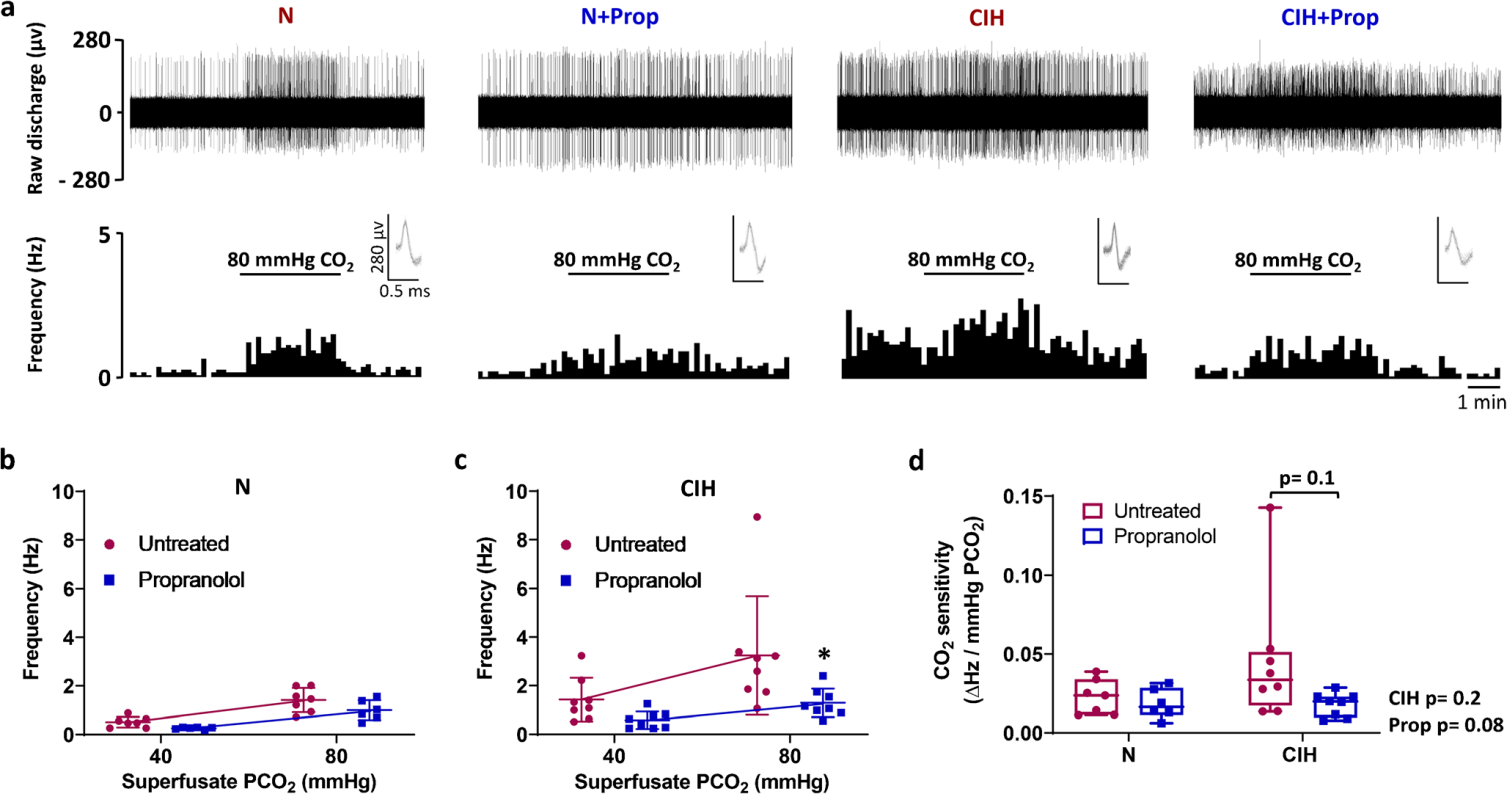
Chronic propranolol treatment reduces carotid body (CB) responses to hypercapnia after chronic intermittent hypoxia (CIH). **a** Example raw traces (upper) and frequency histograms (lower) demonstrating the ex vivo CB chemoafferent response to hypercapnia (PCO_2_ = 80 mmHg) from N, N + Prop, CIH and CIH + Prop animals. For each trace, multiple action potentials are overdrawn shown to exhibit the single fibre discrimination. Mean discharge frequency measured at baseline and in response to hypercapnia for **b** N (*n* = 7) and N + Prop (*n* = 6), and **(c)** CIH (*n* = 8) and CIH + Prop (*n* = 8) animals. Data presented as mean ± SD. * denotes *p* < 0.05 Propranolol vs untreated for CIH animals; two-way repeated measures ANOVA with Bonferroni multiple-comparison test. **d** Mean CO_2_ sensitivity (∆Hz/mmHg PCO_2_) for all 4 groups. Individual points represent averaged data for a single animal. Data presented as box and whisker plots with median; the 25th and 75th percentiles form the box and whiskers extend to minimum and maximum values. Overall effects of CIH and Propranolol are shown as text; two-way ANOVA with Tukey’s multiple-comparisons test

Raw trace examples demonstrating representative chemoafferent responses to hypercapnia for all 4 groups are shown in Fig. 4a. Chronic propranolol treatment significantly attenuated CB chemoafferent activity during hypercapnia in animals exposed to CIH (Fig. 4a–c). Propranolol also tended to reduce CB hypercapnic sensitivity (*p* = 0.1) in the animals exposed to CIH but not N (Fig. 4a–d).

### Propranolol treatment prevents increased vascular sympathetic nerve density evoked by CIH

**Fig. 5:**
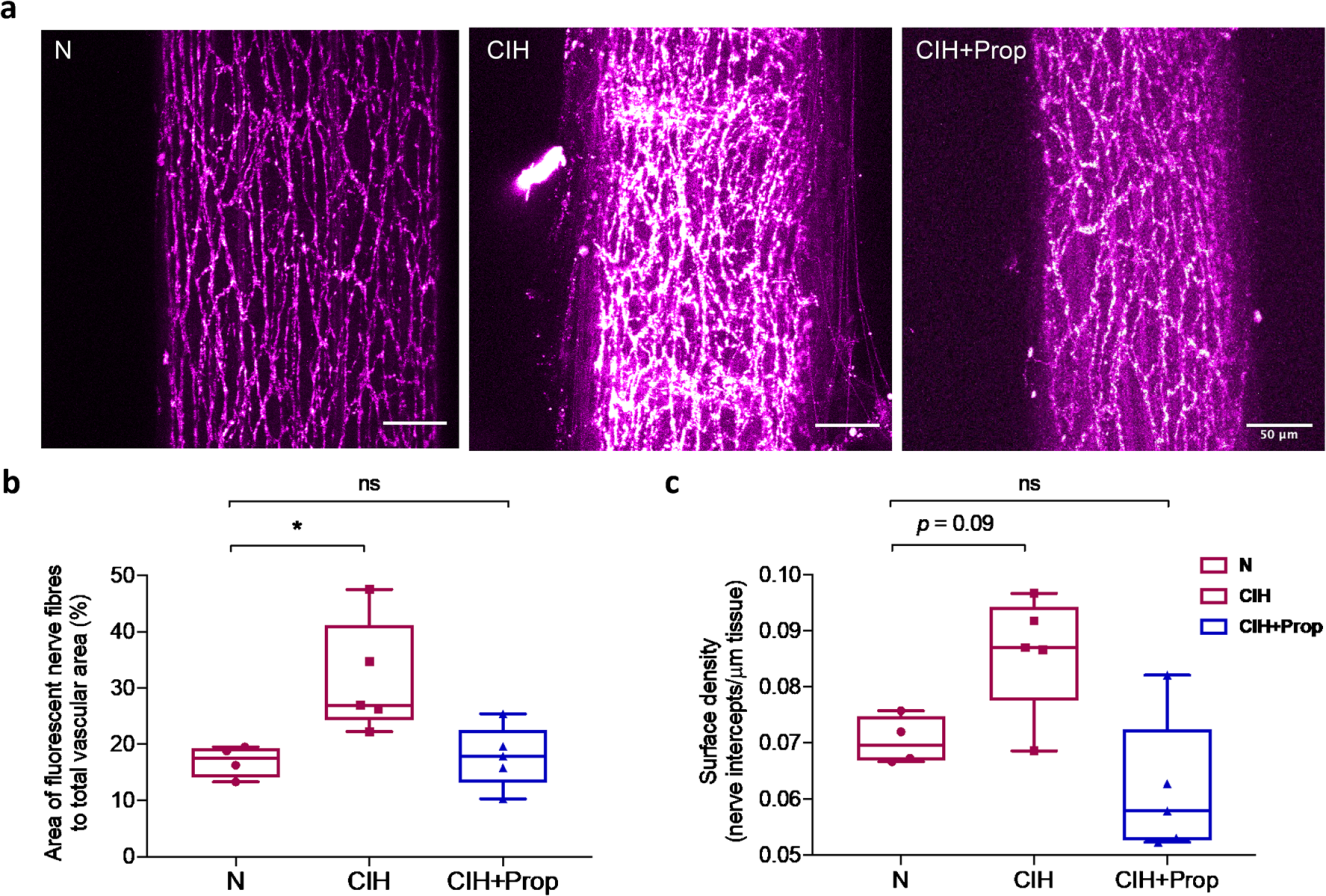
Propranolol treatment prevents the increase in vascular sympathetic nerve density evoked by chronic intermittent hypoxia (CIH). **a** Raw confocal images demonstrating sympathetic nerve fibres expressed on the surface of mesenteric arteries (MA) isolated from N, CIH and CIH + Prop animals. MAs were loaded with a fluorescent dye (Neurotransmitter Transporter Uptake Assay), to reveal noradrenergic sympathetic nerves. **b** Mean percentage of nerve fibre innervation per vessel area in N (*n* = 4), CIH (*n* = 5) and CIH + Prop (*n* = 5) animals. **c** Mean number of nerve fibre intercepts per μm in the same 3 groups. Individual points are averaged data from a single animal. Data presented as box and whisker plots with median; the 25th and 75th percentiles form the box and whiskers extend to minimum and maximum values. * denotes *p* ˂ 0.05 compared to N; ordinary one-way ANOVA followed by Tukey’s multiple comparisons test

To assess changes in periarterial sympathetic innervation in response to CIH and propranolol, we labelled mesenteric artery (MA) noradrenergic nerves with a fluorescent substrate for the noradrenaline transporter (NAT) ex vivo [6]. Merged *z*-stack images taken from MAs of N, CIH and CIH + Prop are shown in Fig. 5a. The percentage of nerve fibre innervation area per vessel was significantly increased in CIH animals (N 17 ± 3% vs. CIH 32 ± 10%, *p* < 0.05, Fig. 5b). Propranolol treatment prevented the increase in innervation area of nerve fibres caused by CIH (N 17 ± 3% vs. CIH + Prop 18 ± 5%, *p* > 0.05, Fig. 5b). In addition, the number of nerve fibre intercepts per μm of tissue was increased approximately by 22% after CIH; however, this was not statistically significant (*p* = 0.09, Fig. 5c). Quantification of single-terminal NAT uptake rate demonstrated no significant differences between N and CIH animals (N: 5 ± 1% min^−1^, *n* = 4 animals, vs. CIH: 5 ± 3% min^−1^, *n* = 4 animals; *p* > 0.05, Welch’s *t* test), suggesting that periarterial sympathetic NAT function is unperturbed by CIH.

### Propranolol reduces respiratory frequency in normoxia and hypoxia

**Fig. 6:**
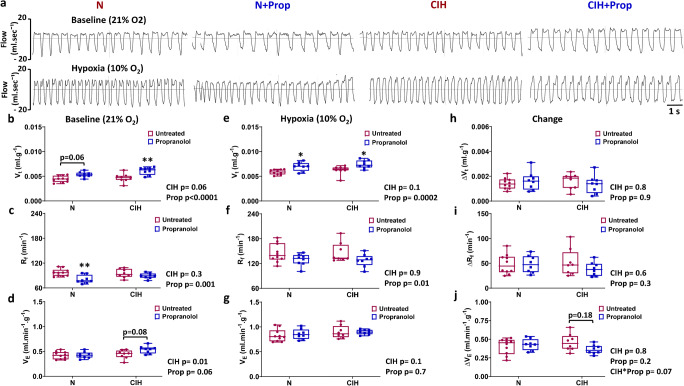
Chronic propranolol treatment alters the respiratory pattern in normoxia and hypoxia. **a** Example plethysmography traces demonstrating 10 s of normoxic ventilation (F_i_O_2_ = 21%, upper) and hypoxic ventilation (F_i_O_2_ = 10%, lower) for N, N + Prop, CIH and CIH + Prop animals. **b**–**d** Tidal volume (V_t_) measured in normoxia and hypoxia, and the calculated difference (hypoxia-normoxia) are shown respectively for N (*n* = 10), N + Prop (*n* = 8), CIH (*n* = 8) and CIH + Prop (*n* = 8) animals. **e**–**g** Respiratory frequency (R_f_) measured in normoxia and hypoxia, and the calculated difference (hypoxia-normoxia). **h**–**j** Minute ventilation (V_E_) measured in normoxia and hypoxia, and the calculated change (hypoxia-normoxia). Individual points represent a single animal. Data presented as box and whisker plots with median; the 25th and 75th percentiles form the box and whiskers extend to minimum and maximum values. Overall effects of CIH and Propranolol are shown as text. *, ** denotes *p* ˂ 0.05, and *p* ˂ 0.01 compared to N or CIH; two-way ANOVA with Tukey’s multiple-comparisons test

The next aim was to evaluate the impact of CIH and chronic propranolol treatment on normoxic and hypoxic ventilation. Propranolol treatment caused a change in the normoxic pattern of breathing as evidenced by a significant increase in V_t_ (Fig. 6a and b) and a significant reduction in R_f_ (Fig. 6a and c), without modifying V_E_ (Fig. 6a and d). This was largely consistent in both CIH and N animals, although post hoc analysis shows that the fall in R_f_ by propranolol treatment was only significant in N animals. In hypoxia, the impact of propranolol was a maintenance of a significantly higher V_t_ and reduced R_f_ without affecting the V_E_ (Fig. 6a–f and g). This effect of propranolol was consistent in both N and CIH animals. Propranolol did not significantly alter the rise in either V_t_ or R_f_ in N or CIH animals in hypoxia (Fig. 6d and g). However, there was a suggestion of an interaction between the CIH and propranolol stimuli on the hypoxic ventilatory response (HVR) (CIH x Prop, *P* = 0.07, two-way ANOVA); this was largely due to the tendency for propranolol to reduce the HVR in CIH but not N animals (Fig. 6j).

### Propranolol reduces blood pressure during hypoxia but not normoxia

**Fig. 7:**
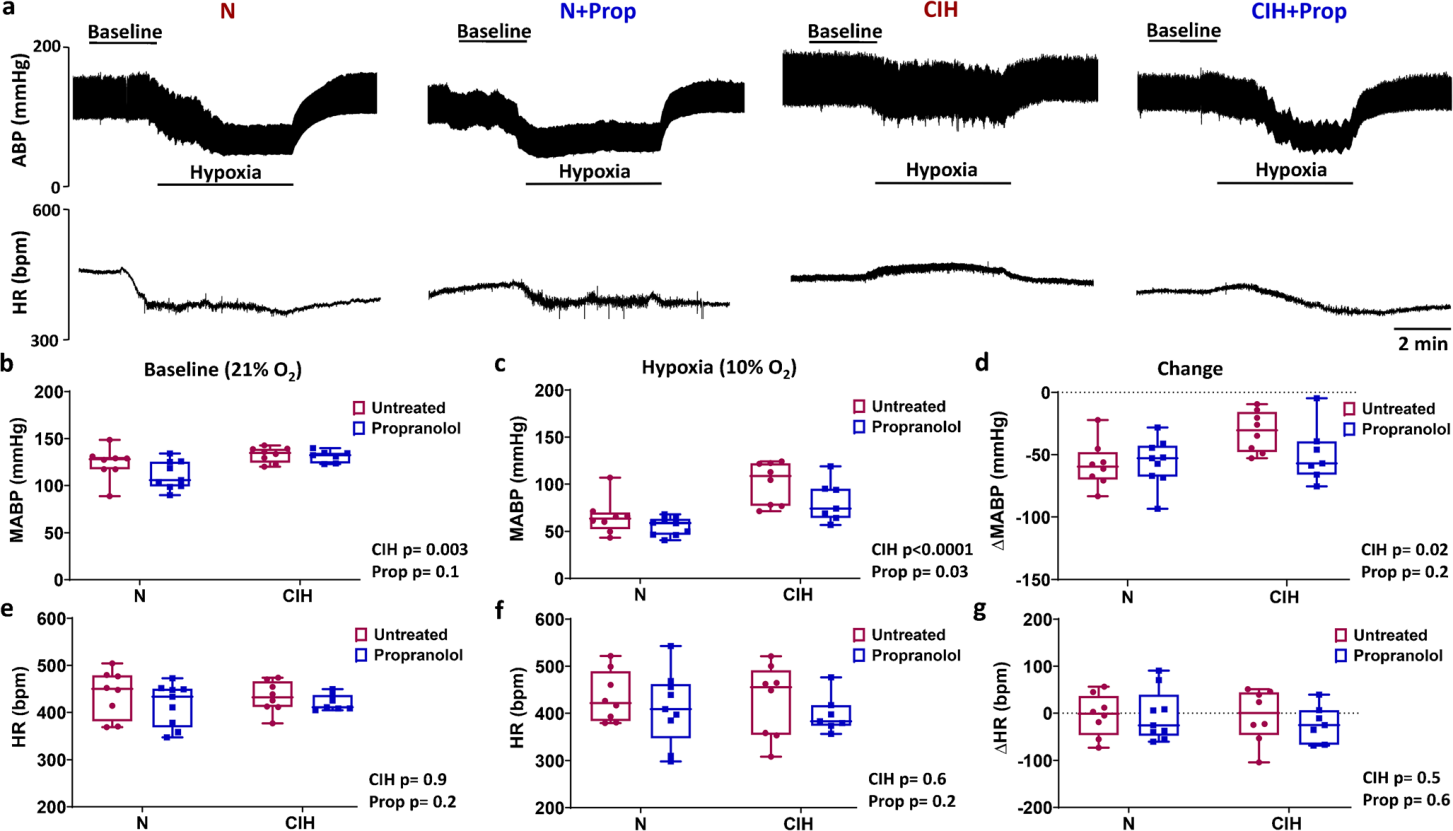
Propranolol reduces mean arterial blood pressure in hypoxia but not normoxia. **a** Example in vivo traces demonstrating 10 min of recording of arterial blood pressure (ABP; upper) and heart rate (HR; lower) during normoxia and hypoxia (10% F_i_O_2_) for N, N + Prop, CIH and CIH + Prop animals. **b**–**d** Mean arterial blood pressure (MABP) measured in normoxia and hypoxia, and the calculated change (hypoxia-normoxia) for N (*n* = 8), N + Prop (*n* = 9), CIH (*n* = 8) and CIH + Prop (*n* = 7) animals. **e**–**g** HR recorded in normoxia and hypoxia, and the calculated difference (hypoxia-normoxia). Individual points are each from a single animal. Data presented as box and whisker plots with median; the 25th and 75th percentiles form the box and whiskers extend to minimum and maximum values. Overall effects of CIH and Propranolol are shown as text; two-way ANOVA with Tukey’s multiple-comparisons test

We next examined the impact of chronic treatment of propranolol on the baseline and hypoxic cardiovascular parameters, and if this was altered following exposure to CIH. As expected, the impact of CIH was an elevation of MABP in normoxia (Fig. 7a and b). In hypoxia, collectively, CIH animals had a higher MABP compared to N (Fig. 7a and d). This was due to attenuation of the hypoxia-induced fall in MABP in CIH animals (Fig. 7a and d). The overall effect of propranolol was a reduction of MABP in hypoxia, but not normoxia (Fig. 7a–c). The action of propranolol was exaggerated in CIH; in 6 out of the 7 CIH + Prop animals, MABP fell by approximately 20% during hypoxia to levels consistent with both N groups (Fig. 7d). In only 1 CIH + Prop animal did MABP fail to fall significantly during hypoxia (Fig. 7d). Neither CIH nor propranolol treatment significantly affected HR in either normoxia or hypoxia in these experimental settings (Fig. 7e–g).

## Discussion

### Main findings

This is the first study to demonstrate the protein expression of β_1_ and β_2_-adrenoceptor subtypes in the CB type I cell. Targeting these receptors with propranolol prevented the CIH-induced rise in baseline CB sensory activity. Chronic propranolol treatment also reduced CB chemoafferent responses to hypoxia and mitochondrial inhibition with nitrite, an effect that was exaggerated in animals exposed to CIH. Interestingly, propranolol abolished the CIH-induced increase in periarterial sympathetic nerve density. Normoxic ABP was not altered by propranolol but it did exaggerate the fall in ABP in the majority of CIH animals during hypoxia. Thus, this study identifies new roles for β-adrenergic signalling in mediating CB hyperactivity, increased vascular sympathetic nerve density and hypoxic blood pressure, in rats exposed to CIH.

### Chronic β-adrenoceptor blockade with propranolol attenuates baseline CB hyperactivity in animals exposed to CIH

Consistent with multiple previous reports, our data demonstrates that exposure to CIH evokes a significant elevation in baseline CB sensory activity [43, 52, 54, 62]. This effect was attenuated by chronic treatment with propranolol suggesting that the rise in baseline CB activity caused by CIH is dependent on chronic β-adrenoceptor activation. Previous work has identified that plasma catecholamines are chronically elevated in both patients and animals exposed to CIH [13, 26, 37, 56, 59], thus providing the chronic stimulus for the CB remodelling. The exact mechanisms underpinning basal chemoafferent AP generation are still unresolved. However, it is probable that overall chemoafferent output in normoxia is due to spontaneous pre-synaptic (type I cell) depolarisation, Ca^2+^ oscillation and neurotransmitter release [27, 76, 77], acting to modulate the frequency of spontaneous APs in post-synaptic fibres triggered due to the presence of a persistent Na^+^ current [9, 10]. It has been reported that β-adrenergic stimulation can augment the persistent sodium current in other cell types [16]. However, given that we identified the majority of β_1_ and β_2_-adrenoceptors in type I cells, it is more likely that chronic adrenergic remodelling acts in part by up-regulating baseline Ca^2+^ fluctuations and neurotransmitter release. Staining for β_1_ and β_2_-adrenoceptors was detected outside of type I cells and so we cannot rule additional actions on type II cells or post-synaptic fibres. Understanding exactly how chronic catecholamines modulate baseline CB chemoafferent activity in CIH will be an important next step.

### β-Adrenoceptor blockade reduces CB chemosensitivity

Interestingly, CIH did not elevate peak or sustained CB chemoafferent responses to hypoxia or hypercapnia. In our experiments, CIH elevated normoxic ventilation but not the response to hypoxia. A heightened chemoafferent output in hypoxia and increased hypoxic ventilatory response after CIH has been reported in some studies [8, 41, 43] but not all [40, 48, 61]. Nevertheless, this was a somewhat unexpected finding. There are a number of reasons that could explain this. First, the CIH frequency and duration used in this study was on the more moderate side, consisting of 8 cycles per hour for 8 h a day which is slightly lower than that used in some other studies [8, 12, 60]. That said, this paradigm was still sufficient to induce hypertension, basal CB hyperactivity and an increase in vascular sympathetic nerve density. Second, it could be that CIH acts to augment CB hypoxic sensitivity rather than the absolute peak response to hypoxia. Third, our studies specifically measured the sensory activity originating from single fibres, whilst the majority of other studies measure whole nerve discharge or multi-fibre activity [8, 60]. It is possible that CIH predisposes to elevated recruitment of additional sensory fibres during acute hypoxia, an effect that would act to increase the total nerve but not single fibre discharge. Investigating AP fibre recruitment, patterning or new growth is an area that remains unexplored in this context and warrants further consideration. However, what our data does suggest, is that rises in single fibre baseline chemoafferent activity, baseline ventilation, vascular hyperinnervation and hypertension occur before the emergence of exaggerated CB and whole body responses to hypoxia.

Propranolol treatment reduced respiratory frequency in hypoxia and this was consistent in both N and CIH animals. Similarly, propranolol reduced sustained CB chemoafferent responses to hypoxia and nitrite, an effect that was heightened in CIH animals. The similar inhibitory effects of propranolol on these two stimuli are perhaps not unsurprising given that hypoxia has been proposed to act via mitochondrial inhibition [2, 5, 11]. This data points towards a novel role of chronic β-adrenergic stimulation in mediating CB remodelling and responses to hypoxia following exposure to CIH. It was also evident that chronic propranolol treatment was more effective in decreasing the hypercapnic chemoafferent activity in CIH compared to N animals, again suggestive of an upregulation in β-adrenergic signalling following CIH. Our previous work has indicated that the acute actions of Adr are to elevate CB CO_2_ sensitivity [3, 38, 72]. What we now show here is that CO_2_ stimulation of the CB after CIH becomes more dependent on the chronic actions of β-adrenergic signalling. Downstream effectors of chronic β-adrenergic stimulation may include cAMP, protein kinase A (PKA) and exchange protein activated by cAMP, all known to acutely modify CB function [45, 47, 65].

G protein signalling has a major role in establishing and modifying CB function [1]. Previous reports have implicated G protein pathways in CIH mediated CB hyperactivity and include 5-HT [53], angiotensin II [32, 36], endothelin [51, 63] and adenosine [67] signalling. We now reveal that enhanced β-adrenergic stimulation contributes to CB remodelling caused by CIH. Adr may well work in unison with these other G protein signalling pathways and mediators, and ultimately converge on the same signalling pathway to impact on the chemosensitivity of the whole organ. An intriguing possibility is that cAMP/and or PKA may act to chronically modify mitochondria function as shown in other tissues [73]. There is evidence that mitochondrial ROS is increased in the CB and other tissues in response to CIH [52, 69]. Precisely how CIH and potentially Adr impact on CB mitochondrial function could be a key area for future consideration, especially given the important suggested role of mitochondria in the CB [2, 5, 20]. It will be of interest to establish the point at which these multiple G protein signalling converge to drive CB remodelling in CIH.

This is the first study to demonstrate protein expression of β_1_ and β_2_-adrenoceptor - subtypes in the CB type I cell. Our previous in vivo studies, using exogenous adrenaline and propranolol, suggest that β-adrenoceptor stimulation of the CB during hypoglycaemia is necessary to activate the CB and increase ventilation to match the concurrent rise in metabolic rate. This is important to preserve PCO_2_ and pH [22, 72]. A key next step will be to evaluate CB sensitivity to adrenaline and hypoglycaemia in animals exposed to CIH. Furthermore, it will be important to examine CB adrenergic signalling in other conditions where plasma adrenaline is persistently raised such as in heart failure and phaeochromocytoma.

### β-Adrenoceptor blockade prevents CIH-induced rises in vascular sympathetic nerve density

CB stimulation induces reflex alterations in autonomic activity to the heart, vasculature and adrenal medulla that collectively modify cardiovascular function. Here we showed that CIH causes an increase in sympathetic nerve density of the mesenteric artery. Previous reports suggest a similar increase in sympathetic nerve density in the tibial and sural arteries following CIH [34]. Thus, there is an emerging body of evidence that CIH induces noradrenergic hyperinnervation in numerous systemic arteries, yet a direct role for this in mediating hypertension is controversial as there may also be a simultaneous reduction in vascular sensitivity to nerve stimulation [34, 71]. Whilst we did not measure vascular sympathetic nerve firing frequencies or reactivity directly, we did demonstrate that CIH does not modify single-terminal NAT function. An important next step will be to evaluate vascular nerve density and function in humans exposed to CIH.

Interestingly, we showed that propranolol protected against this rise in sympathetic nerve density, indicative of a role of adrenergic-induced hyperinnervation. To our knowledge this is the first report to identify that vascular hyperinnervation caused by CIH is dependent on β-adrenergic stimulation. One explanation for this is an indirect consequence of the concurrent reduction in baseline CB hyperactivity. Alternatively, local CIH may itself be promoting the augmentation in nerve growth. Recently, sympathetic nerve development has been suggested to be dependent on tissue hypoxia and HIF1α signalling [4]. It is recognised that CIH leads to HIF1α stabilisation [68], and this could be promoting the observed vascular sympathetic nerve growth. There is also evidence that propranolol and other β-adrenoceptor antagonists (beta-blockers) can interfere with vascular HIF signalling, although a clear mechanism remains elusive [70]. Characterising the exact mechanism of CIH-induced vascular nerve growth and the involvement of β-adrenergic stimulation now warrants a more detailed investigation.

### Propranolol reduces the blood pressure in hypoxia but not normoxia

Despite propranolol reducing CB hyperactivity, vascular sympathetic nerve density and lowering hypoxic ABP, it did not prevent the normoxic rise in ABP in CIH animals. Hypertension induced by CIH is multi-faceted and includes changes in CB function, increased sensitivity of medullary pre-sympathetic neuronal networks [25, 39], endothelial dysfunction [28] and possibly the increase in vascular sympathetic nerve density. Our data suggests that β-adrenergic stimulation accounts for the development of some pathological features caused by CIH but not all. It is possible that a maintained, albeit depressed, input from the CB chemoreceptors is enough to retain the increased output from pre-sympathetic motor fibres originating in the rostral ventrolateral medulla. As bilateral CB ablation carries a high degree of risk both during surgery and thereafter, it may be that pharmacological agents that cause a greater depression in CB baseline activity are required to reduce hypertension in addition to beta-blockers. Interestingly, there have been agents tested which dampen the chemoreflex downstream of the CB in CIH including ibuprofen, losartan, oestradiol and progesterone [7, 24, 42, 64]. Giving beta-blockers such as propranolol alongside one of these agents may be necessary to more effectively reduce cardiovascular disease associated with CIH.

The alterations in CB activity, vascular sympathetic nerve density and arterial blood pressure, without a significant change in the HR in the CIH animals, suggests that the increase in ABP is primarily associated with increased total peripheral resistance. Previous studies have suggested a possible role of baroreceptor stimulation in CIH that acts to counter any rise in HR [31, 33, 75]. This occurs in spite of an overall reduction in baroreflex sensitivity which is itself subject to the severity of CIH paradigm [31, 33, 75]. Although our CIH paradigm did induce alterations in CB and cardiovascular function, we suggest it was not severe enough to alter baroreflex sensitivity and so this mechanism acted to reduce the HR. Furthermore, we did not directly measure changes in cardiac sympathetic nerve density or function in response to CIH, both of which will contribute to HR in normoxia and hypoxia. Measuring cardiac sympathetic innervation density and activity using the novel NTUA assay, as well as evaluating arrhythmia incidence in response to CIH and beta-blockers warrants further investigation.

## Conclusion

The carotid body type I cell expresses both β_1_ and β_2_-adrenoceptor subtypes. In animals exposed to CIH, β-adrenoceptor blockade with propranolol prevents baseline CB hyperactivity, abolishes the rise in vascular sympathetic nerve density and reduces blood pressure during hypoxia. These findings therefore reveal novel mechanisms of β-adrenergic stimulation in evoking CB hyperactivity, sympathetic vascular hyperinnervation and altered blood pressure control in response to CIH.

## Data Availability

Throughout the manuscript, all individual data points are presented and indicate averaged data from a single animal. Therefore, all data generated or analysed during this study are included in this published article.

## Abbreviations

CB: Carotid body
MABP: Mean arterial blood pressure
HR: Heart rate
V_E_: Minute ventilation
V_t_: Tidal volume
R_f_: Respiratory frequency
HVR: Hypoxic ventilatory response
CIH: Chronic intermittent hypoxia
OSA: Obstructive sleep apnoea
MA: Mesenteric artery
